# Assessment of mudstone compaction in exploration wells in the Rovuma Basin, offshore Mozambique

**DOI:** 10.1016/j.heliyon.2019.e02641

**Published:** 2019-10-21

**Authors:** Oscar J. Nhabanga, Philip S. Ringrose

**Affiliations:** Norwegian University of Science and Technology (NTNU), Norway

**Keywords:** Geology, Geophysics, Petroleum engineering, Petrology, Lithology, Mineralogy, Mudstone, Rovuma basin, Sonic transit time, Pore pressure, Effective stress, Resistivity

## Abstract

In order to develop an improved pore pressure prediction model for the overburden mudstones in the Rovuma Basin, offshore Mozambique, we apply Eaton's method to example well data from three exploration wells, which intersect Quaternary, Tertiary and Cretaceous sediments over depth intervals down to ∼3 km below seafloor. The predictive method only included the effects of mechanical compaction, which is a reasonable assumption for the low-temperature shallow sections. We found that Eaton's method applied to resistivity and acoustic log attributes works well and can be used to identify the mudstones that display over-pressured or normally pressured sections. The predicted pore pressures showed a good match to pore pressures in permeable formations. Using this calibration, we derived an improved pore-pressure prediction method for these wells and for the Rovuma Basin in general. The resulting model should give a good basis for future analysis of compaction processes and pore pressure in this basin.

## 1 Introduction

The Rovuma Basin is located in East Africa mainly in northern Mozambique. It covers both offshore and onshore sections and contains important petroleum resources (Davison and Steel, 2018). The basin is characterized as a passive basin margin, but with a complex tectonic history following the formation of the East Africa rift system in the early Triassic followed by episodes of rifting and inversion in the Late Jurassic to Cretaceous (Macgregor et al., 2018). This relatively under-explored basin presents new challenges for the interpretation of petrophysical data from exploration wells as a basis for field development.

Here we use well logs to discriminate the lithology, and then we detect and predict overpressured mudstones in three exploration wells from the offshore Rovuma basin and finally we establish the compaction trendline for each mudstone interval, which is the porosity change through depth for mechanically compacted mudstone.

A major challenge for deep-water wells is related to overpressure, where drilling of high-pressure sediments may cause significant time delay in drilling, due to excessive pressure kicks, borehole instability, or stuck pipe incidents, and may even cause a complete loss of the well if not accurately predicted (Zhang, 2011; El-Werr et al., 2017). In some cases, drilling overpressured sediments has been reported to induce geological disasters (Davies et al., 2007).

Pore pressure, which is defined as the pressure of the fluid within the pore space of the formation, is usually equivalent to hydrostatic pore pressure, if there is a connected pore system. Pore pressure higher than the hydrostatic pressure is referred to as overpressure, or simply abnormal pore pressure, and is usually caused by a permeability restriction. Overpressure in shales is mainly caused by disequilibrium compaction, which is a rapid burial of mudstone, where the pore fluid is not given enough time to escape (Zhang, 2011).

Pressure measurements in shales are usually unavailable due to their low fluid mobility, and so the model performance cannot be evaluated directly. Therefore, we compare the methods used to predict pore pressure with well pressure measurements obtained in adjacent permeable sandstone or carbonate formations (as done by others, e.g. Alixant and Desbrandes, 1991).

A summary of burial history of the Rovuma Basin is provided to help understand the compaction trends of mudstone and possible overpressured mudstone layers. We additionally discuss the formation temperature variation in order to assess the compaction regime of the mudstone.

### 1.1 Burial history of the Rovuma Basin

**Fig. 1:**
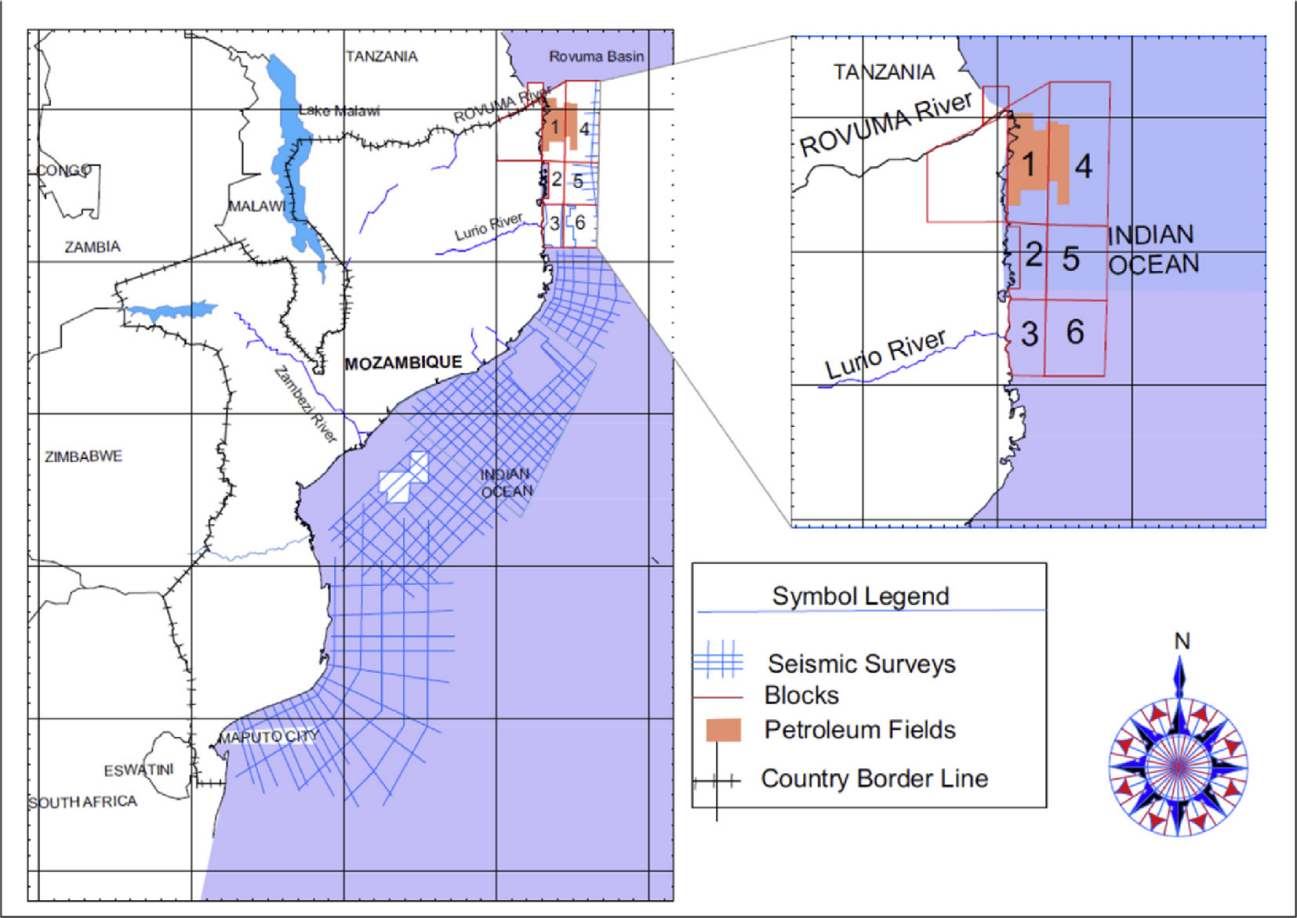
Regional map showing seismic data in Mozambique (Seismic lines from Francis et al., 2017).

This study covers three exploration wells, namely Buzio, Cachalote and Dugongo located in the Rovuma Basin (RB) offshore Mozambique, in exploration blocks 2 and 4, as shown in Fig. 1.

Several researchers, such as Salman & Abdula (1995) and Mahanjane and Franke (2014), argue that the basin sequence starts with the Karoo formation at top basement level. Summarizing the work of Salman and Abdula (1995) and Francis et al. (2017), we can identify the most important features of the basin sequences as follows:

• The top of the main syn-rift sequence is represented by strata of the Lower to Middle Jurassic period, where there is a development of marine shales, carbonates and sandstones with several unconformities.
• The Upper Jurassic marks the start of the main post-rift section, with deep-water deposition of marine shales and some sandstones (Salman and Abdula, 1995; Mahanjane and Franke, 2014). These deep Jurassic shales are believed to be the main source rock for the abundant hydrocarbons in the Rovuma Basin.
• During the Upper Aptian (Lower Cretaceous), some important deep-water sandstones were deposited, caused by falls in sea level and the influx of sand sheets from the exposed upper parts of the basin. On the western side of the basin, the Lower Cretaceous sediments are directly observed on top of pre-Cambrian basement and are exposed as immature continental conglomerates and quartz-feldspathic sandstone.
• The Upper Cretaceous deep-water deposits occur as a sequence of marls, argillites and considerable amounts of gypsum (Salman and Abdula, 1995). Francis et al. (2017) also point out that the Upper Cretaceous is typically characterised by low deposition rates.
• Reactivation of tectonic controls giving higher sedimentation rates due to enhanced onshore uplift and pulses of deep-water deposition characterize the thick Tertiary sequences. Sediments of Paleocene to Early Oligocene age are mainly characterized by shallow-water facies.

Edwards and Lainchbury (1999) and Francis et al. (2017) argue that the highest burial rates (>0.01 mm/yr) occurred during the Oligocene to Miocene, related to East Africa rifting episodes in that period. These high burial rates drove the source-rock intervals into the gas window. The Miocene deposits are the thickest of the Tertiary Period and are represented by marine shales and sandstones (Salman and Abdula, 1995; Mahanjane and Franke, 2014). The Quaternary Period is marked by a thinner, passive-margin sequence with lower rates of sedimentation.

**Fig. 2:**
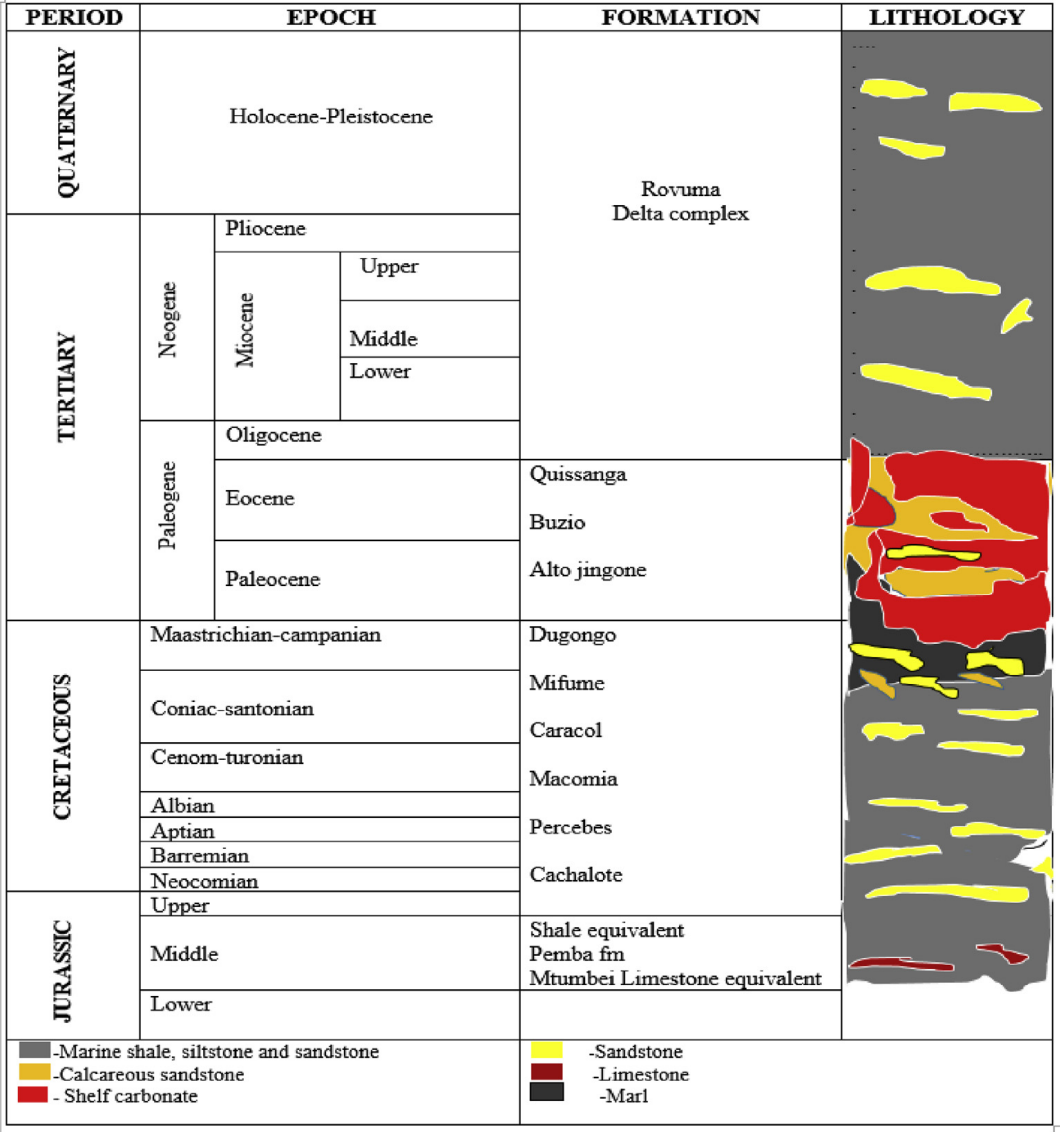
Summary of Rovuma Basin stratigraphy offshore Mozambique (Modified from Key et al., 2008 and Mahanjane and Franke, 2014).

Fig. 2 summarizes the offshore stratigraphy of the Rovuma Basin showing some of the formations evaluated in this study, which covers an interval from the Jurassic through to the Quaternary Periods. The exploration wells analyzed are Buzio-1, Cachalote-1 and Dugongo-1. The Buzio-1 (Bu-1) well intersects the Buzio, Pemba and Percebes Formations, while the Cachalote-1 (Ca-1) well intersects slightly older sequences from the Cachalote Formation through Pemba (hard limestone) Formation and ending with the Cachalote and Caracol formations. Both wells are in Block 2. The Dugongo-1 (Du-1) exploration well in Block 4 intersects only the Dugongo Formation. Although the Cachalote-1 well intersects older formations than the Buzio-1 and Dugongo-1 wells, it is shallower due to its structural position. The three wells thus give a good, although limited, insight into the main stratigraphic sequences and the effects of burial to different depths. In future, further wells could be included, but they were not available for this study.

### 1.2 Determination of lithology and geothermal gradient

Discrimination of lithology is a common activity in the petroleum industry. Prediction of petrophysical properties requires a robust knowledge of the lithology, and at the well site, lithology discrimination is usually performed by interpreting the drill cuttings, which is the cheapest way, but improved lithology determination can also be made using core samples. Another method is to use the well logs to give a general expectation of lithology, also called electro-facies analysis. However, this is not an absolute discriminative method for lithology, and drill cuttings or cores are required for confident interpretations.

**Fig. 3:**
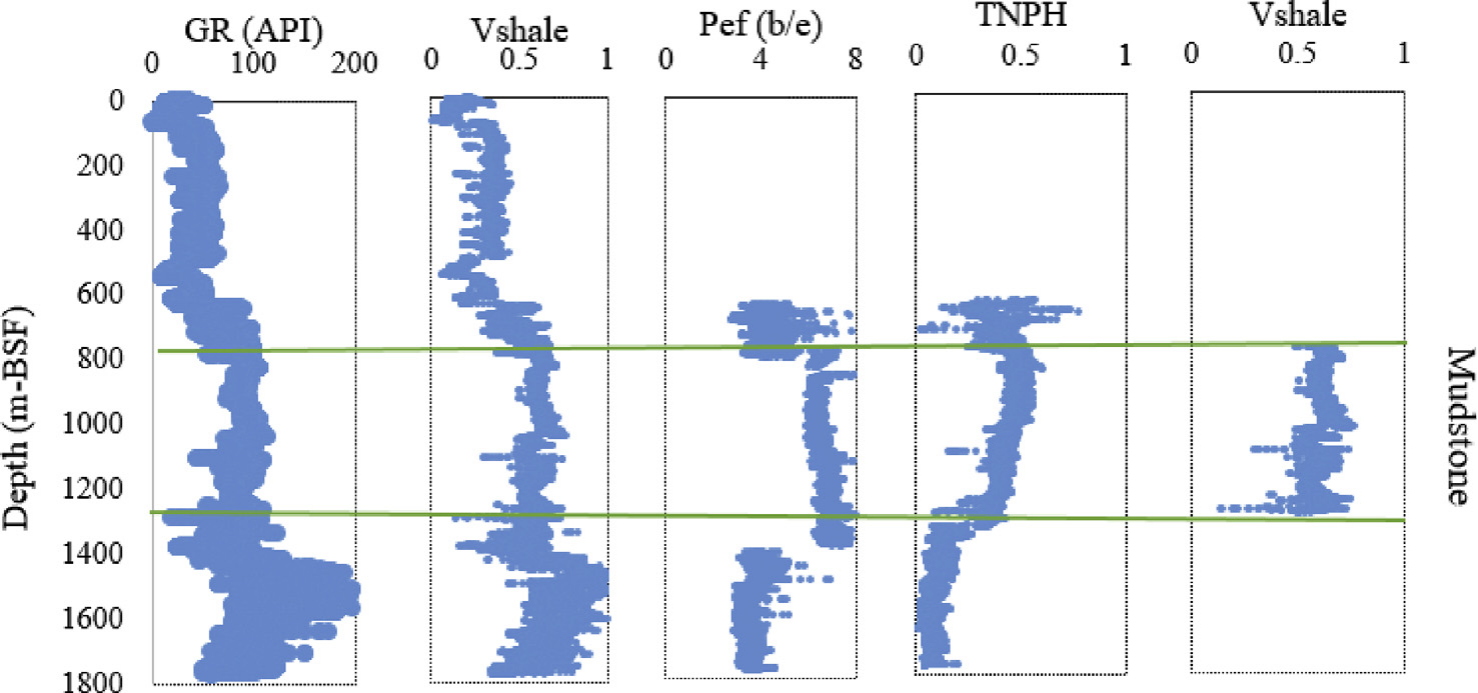
A typical example of raw data (GR, Vshale, Pef and TNPH logs), and Vshale of discriminated mudstone section plotted as a function of depth from the Cachalote well.

Using standard log-based lithology discrimination methods, such as gamma ray (GR), shale volume (V_shale_), photoelectric absorption (P_ef_), and neutron (TNPH) logs, together with the aid of well reports, we identified the main lithology groups as shale (mudstone), sandstone, and limestone. Shale volume (from gamma ray logs), neutron, and P_ef_ logs were the main lithology indicators, and by integrating them, we were able to improve the lithology discrimination significantly (by reference to well reports). Fig. 3 provides a typical lithological discrimination of the Cachalote well mudstone, where based on the log availability, the mudstone section was discriminated. A similar process was followed for the Buzio and Dugongo wells. The applied cut-offs are given in Table 1.

**Table 1:** Cutoffs applied for lithology discrimination.

Lithology	V_shale_ (%))	Neutron Porosity (%)	P_ef_ (b/e)
Sandstone	<35	<30	1.6–1.8
Limestone	<50	<20	4.5–5.08
Shale	>60	>30	>3

Possible misinterpretation of shale volume could occur in feldspathic sandstones, which present high V_shale_ (high gamma ray) readings, but this problem was resolved by contrasting the results with the Neutron and P_ef_ logs, because feldspathic sandstones have lower neutron porosity and lower P_ef_ values than shales (mudstones). However, some difficulties persisted, for example, in shaly-limestone intervals due to high shale volume in the in Buzio well. Well reports therefore played an important role as additional verification tools for lithology discrimination.

The geothermal gradient is also a key parameter, since the formation temperature affects the logging tools, the drilling muds, and helps predicting the type of expected hydrocarbon, the compaction regime, as well as the expected porosity and permeability ranges. Most importantly, higher temperatures are associated with the onset of chemical compaction (Bjørlykke, 1999; Bjørkum et al., 1998). The offshore geothermal gradients of the three wells were estimated up to maximum drilled depth by using a linear trend, as given in Table 2. The geothermal gradients of the Buzio-1 and Cachalote-1 wells are similar and significantly greater than the Dugongo-1 well. The large difference in geothermal gradients between Dugongo-1 and the other two wells is most likely either due to effects of crustal heat flow related to the tectonic position in the wells, but could also be influenced by subsurface aquifer flows.

**Table 2:** Estimated geothermal gradients and maximum depth temperature.

Well names	Geothermal gradients (^°^C/100 m)	Maximum depth temperature (^°^C)	Maximum drilled Depth from the Seabed (m)
Buzio-1	5.46–5.50	103	1796
Cachalote-1	4.88–4.92	92	1778
Dugongo-1	3.32–3.34	112	3186

High temperatures can also contribute to causing of abnormally-pressured mudstones by promoting cementation; however, abnormal pressures may occur at low temperatures, especially in settings with high sedimentation and burial rates. In our case, the stratigraphic intervals and tectonic settings with high sedimentation rates mainly occurred in the Cretaceous and Tertiary sequences (Oligocene-Miocene series). In these settings, if fluid escape was sufficiently restricted by mudstone units due to their very low permeability, then high pore pressures would be expected. We therefore focus on evaluating the mudstone compaction as the primary control of overpressure.

### 1.3 Pore pressure prediction methods

A key requirement for safe well planning in all lithological formations is accurate pore pressure prediction. There are several methods for predicting pore pressure and the list continues growing. Ideally, all of them are supposed to give similar results, but different method assumptions and basin settings vary, making some methods better that others.

In many offshore basins around the world, pore pressure has been successfully predicted based on the principles of mechanical compaction (Eaton, 1975; Bowers, 1995; Kumar et al., 2006) and chemical compaction (Bowers, 1995; Ramdhan and Goulty, 2017). However, it was initially unclear to what extent these models can be applied to this basin.

Among several pore pressure prediction methods developed to understand the sedimentary basins under mechanical compaction (Bjørlykke, 1999; Dewhurst et al., 1998), we summarise the principles of the most relevant, providing the advantages and disadvantages, and the reasons they may or may not be applicable for the Rovuma basin.

The common assumption in all these methods is that the primary mechanism that causes overpressure is disequilibrium compaction (Zhang, 2011). The main differences in prediction results are related to the compaction mechanisms assumed, or the effects of hydrocarbon generation and aqua-thermal expansion, or the assumptions used in generating the correlations, and lastly the lithology of the formation.

Many pore pressure methods are based on establishing a normal compaction trendline (NCT) versus depth. The main assumption is that the NCT can be established as mudstone compacts with depth, with pore pressure remaining equal to hydrostatic pressure. Methods using NCT include the ratio method and the Eaton's method, discussed below. When pore pressure is caused by more than one mechanism, a method such as Bower's method is recommended (Bowers, 1995; Nygaard et al., 2008). Methods such as Alixant and Desbrandes (1991) and Holbrook (1994) do not use a normal compaction trendline, but still assume that disequilibrium compaction is the primary compaction mechanism.

The ratio method (Mouchet and Mitchell, 1989) is applied using the principle that the difference between the observed well log data and a normal trend (NCT) are proportional to the pressure increment. The method is simple to apply, but the disadvantage is that the predictive isodensity lines are solely valid for the specific overpressure condition of the well they were derived for, and sometimes the results can be very unrealistic with pore pressure values greater than the lithostatic stress.

Another approach is the Alixant method (Alixant and Desbrandes, 1991) which uses shale resistivity and temperature data, along with the effective stress estimate for pore pressure prediction. However, experimental or published laboratory constants are required, needing access to a core dataset. The advantages of this method are that it does not require a normal compaction trendline (NCT) and that analytical relationships are derived using meaningful calibration coefficients. This method shows some disadvantages in case of limited core data, but may be useful in mature stages of field development.

The Holbrook method (United States of America Patentnr. 5,282,384, 1994) is potentially useful for pore pressure prediction. A log of lithostatic stress is generated from the directly measured lithostatic stress in the well. The maximum effective stress and compaction exponent for a specific mineral are determined from linear relationship between the logarithms of effective stress for a specific mineral and solidity. The method can accurately predict the pore pressure, and the main advantage is that usually there is no need for additional costs after the well is drilled. Some disadvantages are that it is mainly valid for high porosity lithologies, that it requires minerals (or group of minerals) to be specificied through depth for accurate effective stress prediction, and finally that it does not work well for deeper wells with low porosity caused by high effective stress.

A more promising and widely-known method for pore pressure prediction is the Bower's method (Bowers, 1995), which can be used for both loading and unloading processes (i.e. burial and uplift). The method relies solely on sonic velocity data. It introduces for the first time a distinction between overpressure caused by disequilibrium compaction and by fluid expansion. The parameters involved require some calibration from core or from seismic and well data, such as offset velocity data and effective stress data. The unloading curve is empirically derived and correct values for unloading parameters are needed. The method is difficult to apply in a frontier basin setting with limited wells. However, if enough data are available, the Bower's method is a very powerful method for pore pressure prediction.

The Eaton's method (Eaton, 1975) uses the NCT curve and is very versatile, simple and easy to apply. It has been widely used for young sedimentary rocks, and it works better for the setting of exploration wells in both the planning phase and during drilling operations. Its application relies on having velocity and resistivity data, density and d-exponent estimates from well logs. Originally, it was designed for pore pressure prediction under the disequilibrium compaction mechanism, but it proved to be valid for sediments under fluid expansion mechanism (Bowers, 1995). In general, the method does not take into account the unloading process. Recent work demonstrating how Eaton's method can be applied was pulished by Zhang (2011).

Eaton's method fails to predict accurately the pore pressure if the wrong NCT is defined, or when there are few data points to define it, or perhaps in older sedimentary rocks. Additionally, it may give erroneous results if the NCT is defined over an interval that is already overpressured, therefore underestimating overpressured intervals, where the final output could be a false pressure estimates which may pose risks during drilling.

In this study, we use the Eaton's method to predict pore pressure, using the available well log data and carefully taking into account the age and temperature of the sedimentary formations. We conclude that Eaton's method is suitable and can be successfully applied for pore prediction in this basin, with some constraints.

## 2 Theory

Compaction of granular sedimentary rocks is influenced by deformation caused by the balance between the external stress and the pore pressure. The compaction of sediments that occurs at low temperatures, before the onset of diagenesis, is dominated by mechanical compaction and is primarily driven by the effective stress. As diagenesis begins, the compaction also becomes influenced by temperature-controlled chemical processes. This causes dissolution and precipitation of rock, and mainly causes cementation (leading to very low porosity rock). In diagenetically-influenced mudstones, pore pressure may be underestimated, if mechanical-compaction models is assumed, because the effective stress may be overestimated (Ramdhan and Goulty, 2017).

The temperature for significant onset of cementation generally occurs in the range of 70–100 °C (Bjørlykke, 1999; Storvoll and Brevik, 2008). The effective stress ($\sigma_{e}$), which essentially controls mechanical compaction, is put most simply as the difference between the overburden stress and the pore pressure, as shown in Eq. (1):

(1) $$\sigma_{e}=\sigma_{v}-p_{p}$$

Here we ignore lateral stresses and only take into account the net vertical stress, since it is the dominant contribution to compaction in a mechanical regime (Yang and Aplin, 2004; Alixant and Desbrandes, 1991).

The degree of compaction depends on rock type. Different lithologies will have different compaction rates and minimum porosity ranges. Although mudstones have the highest porosity at deposition time, the porosity decreases rapidly to become the lowest residual porosity under mechanical compaction, in contrast to sandstones, which resist compaction and maintain a relatively high porosity during burial.

In normally compacted mudstones, compaction is a steady-state process, meaning that the porosity is reduced as a steady function of the effective stress. However, abnormally compacted mudstones are revealed by having a higher porosity at a given depth. Rapid burial of mudstones is usually the main cause of overpressured mudstones, but chemical compaction caused by the diagenesis of clay minerals may also contribute (El-Werr et al., 2017). Finding these abnormal mudstones intervals is a key to identifying zones of overpressure.

It is important to know the pore pressure of the formation before or during the drilling process, because it helps avoiding drilling in underbalanced conditions, meaning that the wellbore pressure is lower that the formation pressure, which may lead to high pressure kicks, borehole instability and possibly uncontrolled blow outs with consequent loss of the well. Pressure prediction also helps avoiding drilling with extremely overbalanced mud-weight conditions, meaning that the drilling pressure is conducted at pressures higher than necessary, which may cause damage of the formation, such as fracturing the formation (causing mud loss) or creating reduced permeability due to excessive mud invasion of permeable formations.

### 2.1 Eaton's method

Here we settled on our preferred approach of using Eaton's method. This method is aimed at establishing a relationship between well log data, such as deep resistivity and acoustic data, using effective stress theory. The procedure is to examine the relationship between vertical stress and vertical depth (TVD) and to estimate the ratio between log data and a normal compaction trend (NCT). Eaton's method has proven to be useful and robust for many exploration wells, and it relates changes in pore pressure to departure from normal log attributes. The underlying assumption of Eaton's method is that a ratio of log values may be obtained by comparing regions of normal and abnormal pressure for the region of interest (Kumar et al., 2006; Zhang, 2011).

As a rule of thumb, it has been argued that empirical methods for pore pressure prediction do not work for mudstone temperatures greater than around 70 °C, due to pronounced effect of chemical compaction (diagenesis), because that will tend to remove mudstones from their position on the NCT (Ramdhan and Goulty, 2017; Swarbrick, 2002). However, that point varies from basin to basin. Other conditions, such as a source of potassium (e.g. feldspathic mineral) and the burial time also influence the rate of chemical compaction.

Cementation generally reduces the porosity relative to normal compaction, and if effective-stress methods for pressure prediction are used in this compaction domain, the pore pressure may be underestimated. Ramdhan and Goulty (2017) proposed a method to predict pore pressure where chemical compaction plays an important role, at temperatures of around 100 °C. This effect was not considered in our study where the temperatures are generally lower than 100 °C, but the possible effect of chemical processes is important to bear in mind, especially for the deeper and hotter intervals.

In general we found that Eaton's method works well for pore pressure prediction in the Rovuma Basin mudstone intervals studied here, where mechanical compaction is expected to dominate.

We summarize Eaton's approach using Eqs. (2), (3), and (4). Pore pressure, P_p_, is defined by:

(2) $${\text{p}}_{\text{p}}=\sigma_{\text{v}}-\left(\sigma_{\text{v}}-{\text{p}}_{\text{h}}\right)\cdot {\left(\frac{{\text{A}}_{\text{obs}}}{{\text{A}}_{\text{norm}}}\right)}^{\text{x}}$$

Where (A_obs_/A_norm_)^x^ is a calibration term for matching the effective stress estimate to the well log function. We assume that A_obs_/A_norm_ is the ratio of observed log data and the normal trend. In our analysis we used exponents of x = 1.2 for the resistivity logs and x = 3 for the sonic velocity logs as recommended and found this worked generally well.

We then estimated the hydrostatic pressure, p_h_, by integrating the water (brine) density from the surface reference point, D_KB_.

(3) $${\text{p}}_{\text{h}}=\text{g}\cdot \int_{0}^{\text{z}}\rho_{\text{Water(D)}}\mathrm{d}z=\text{g}\cdot \left({\text{D}}_{\text{KB}}-\text{KB}\right)\cdot \rho_{\text{water}}+\text{g}\cdot \int_{\text{BSF}}^{\text{z}}\rho_{\text{Water}}\mathrm{d}z$$

The overburden stress is then estimated by integrating the bulk density from well log as follows:

(4) $$\;\sigma_{v}=g\cdot \left(D_{KB}-KB\right)\cdot \rho_{water}+g\cdot \overset{z}{\underset{BSF}{\int }}\rho_{\text{log}\left(D\right)}\cdot \mathrm{d}z$$

It is assumed that the brine density is constant, and the effect of well inclination is insignificant.

Here we focus on the improved understanding of mechanical compaction in mudstone units within this basin, by assessing the applicability of Eaton's method, and using it to evaluate the implications for prediction of pore pressure. The resulting porosity vs. effective stress functions were then compared with empirical functions and laboratory data to check the validity of the mechanical compaction models and the identified zones of overpressure.

### 2.2 Compaction fitting lines

Compaction of rocks can be summarized by a trendline of porosity change through depth, which is often best expressed as porosity change as a function of effective stress.

This trend helps understanding the degree and the type of compaction of different lithologies. Mechanical compaction is expected to be primarily dependent on effective stress while chemical compaction is expected to be revealed by departures from the effective-stress trend. Several rock compaction studies have been published; however, each rock type has a specific compaction trend, where lithology type and composition, depositional time, burial rate and temperature all play an important role.

We investigated the compaction trends of mudstones in the Rovuma Basin, offshore Mozambique by plotting porosity variation as a function of effective stress in the three exploration wells studied (Fig. 5). Resistivity and acoustic velocity log data were used to estimate pore pressure, and consequently the effective stress. Total porosity (ϕ_total_) values are derived from the Archie's equation using shale resistivity (R_sh_) and assuming 100% water saturation, with an average water resistivity (Rw) from well logs of 0.06 Ω m. The simplest form of total porosity is given as follows in Eq. (5).

(5) $$\phi_{total}=\sqrt{\frac{R_{w}}{R_{sh}}}$$

## 3 Results

**Fig. 4:**
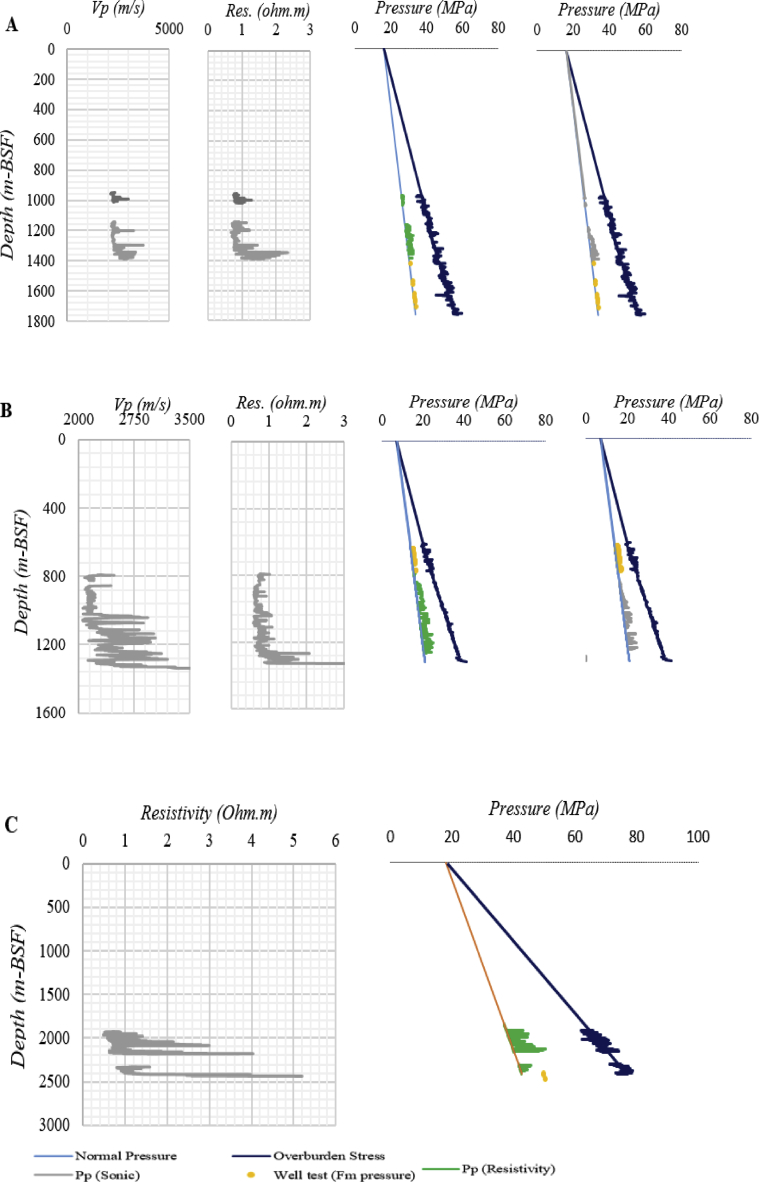
Predicted pore pressure in Paleogene and Cretaceous mudstone units using resistivity and velocity data, and measured formation pressure (well test) in (A) Buzio, (B) Cachalote, and (C) Dugongo exploration wells in Paleogene and Cretaceous mudstones.

We estimated the pore pressure in the Paleogene and Cretaceous mudstone sections using two well logs (deep resistivity and compressional acoustic velocity) for three exploration wells in offshore Rovuma Basin (Fig. 4). These logs were analysed to search for mudstone intervals with either normal or abnormal pore pressure, using the assumption that for normal pressured mudstones, the resistivity and acoustic velocity logs increase with depth, while for the abnormally pressured mudstones, the velocity log can decrease with depth due to unloading, therefore departing from the normal compaction trendline.

The *in situ* temperature is important in this analysis since at higher temperatures chemical compaction will begin to have a significant influence. The estimated temperature range for the Cretaceous mudstone in the Cachalote well varies between 45-72 °C, while for the Buzio well it ranges between 58-62 °C for the Paleogene mudstones and between 71-84 °C for the Cretaceous mudstones. For the Dugongo well, the Eocene mudstone had temperature ranges of 65–76 °C while the Paleocene mudstone was in the range of 81–85 *°*C.

As a general principle, we assumed that chemical compaction effects are likely to become significant at around 70 °C (Bjørlykke, 1999). It is therefore likely that the Cretaceous mudstone intervals in the Cachalote well and the Paleogene mudstone intervals in the Buzio well are dominated by mechanical compaction processes. However, the deeper mudstone intervals in Dugongo well and the Cretaceous mudstone in the Buzio may be affected by chemical compaction.

The water depths for the Cachalote, Buzio and Dugongo exploration wells are 692 m, 1563 m, and 1807 m, respectively. Hydrostatic pressure was estimated by assuming a brine density of 1.05 g/cc in entire drilled depth from below seafloor; however, it is known that the brine density often increases slightly with depth but the effect is small and the predictions will not be affected significantly. Pore pressure in the mudstone sections was then estimated for each well using the Eaton's method for deep resistivity and sonic velocity logs: we then compared our estimates to the available well test data obtained from permeable formations near the mudstones under study. The results are given in Fig. 4.

The Buzio well has two distinct mudstones, the Paleogene (upper) and Cretaceous (lower), as shown in Fig. 4 (A). The Paleogene mudstone is expected to be normally compacted, since velocity and resistivity logs increase gradually with depth. In the Cretaceous mudstone, acoustic velocity and resistivity logs remain almost constant between 1180 m and 1300 m depth. Suggesting that the mudstone could be overpressured in this interval.

Given the estimated temperature range, the Cretaceous mudstone could be somewhat affected by chemical compaction. The predicted pore pressure (by Eaton's method) shows normal pore pressure in the Paleogene, but a slightly overpressured mudstone in the Cretaceous mudstone. This was observed for both the resistivity- and acoustic-log cases, Fig. 4 (A), suggesting the effect is real. Although chemical compaction may have started for the Cretaceous mudstone interval, mechanical compaction is suspected to be predominant.

In Cachalote well (Fig. 4 B) for the Cretaceous mudstone, we observed that at depths between 800 m and 1000 m, the velocity and resistivity logs are almost constant, but below that, the acoustic velocity increases while resistivity shows no significant changes. This suggests that the mudstone could be overpressured. The estimated temperature range implies that the mudstone compaction is controlled by mechanical compaction rather than chemical. However, the pressure estimation as a function of depth shows only slightly elevated pore pressures, for both the resistivity- and velocity-log cases.

Only resistivity logs were available for the analysis of the Paleogene (Eocene and Paleocene) sequences in the Dugongo well (Fig. 4 C). No significant depth trend in the resistivities below 2100 m depth was apparent albeit with significant fluctuations. The deeper Paleocene interval in the Dugongo well is also expected to be more affected by chemical compaction, and the predicted pore pressure may well be underestimated. Based on our estimation, we suspect an over-pressured mudstone.

Application of Eaton's method in these well thus reveals a clearly over-pressured formation in the Cretaceous mudstone at the Buzio well and slightly elevated pore pressures in the Cretaceous mudstone at the Cachalote well. For the Paleogene mudstone we find a clear absence of overpressure in the shallower Buzio well, but some indications of elevated pore pressures in the deeper Dugongo well (albeit with high uncertainty).

To give some validation of this interpretation, we compared our log-based analysis with available pressure measurements from well-test data in adjacent sandstone units (shown in Fig. 4). These sandstone pressures are not expected to match mudstone pressures, but give an indication of depth trends. Shallow Paleogene well tests reveal pressures close to hydrostatic pressure (Fig. 4A and B); while the deeper Paleogene sandstone pressures (Fig. 4C) show distinctly overpressured sandstones. These measured sandstone pressures therefore appear to confirm the mudstone pressure trends derived from well-log analysis.

We then looked at the porosity trends to see if this could confirm the inferences made from pore-pressure analysis.

**Fig. 5:**
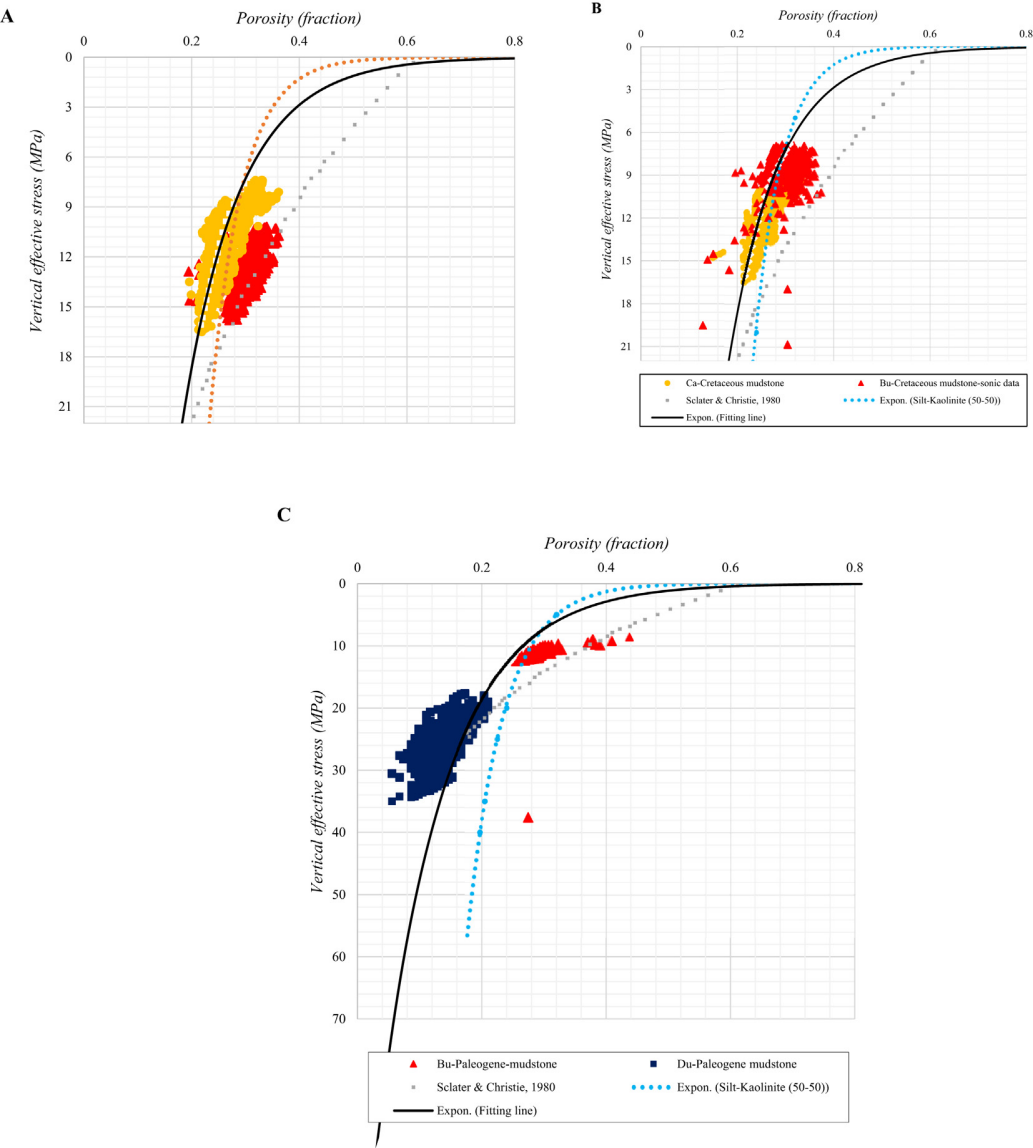
Porosity variation as a function of vertical effective stress for Cretaceous mudstones in the Buzio well (A) and the Cachalote well (B) using resistivity and acoustic data. The same analysis for the Paleogene mudstone (C) in the Buzio and Dugongo wells uses only the resistivity log. Data are compared with functions proposed by Sclater and Christie (1980) and experimental data for a 50-50 silt-kaolinite mix from Mondol.

The analysis of the Cretaceous mudstone*s* (Fig. 5 A and B) shows a maximum (shallow) porosity of about 36% and minimum of about 20%, while the effective stress varies between 6 and 21 MPa. The observed porosity follows the expected trends for mechanical compaction (although the Cretaceous mudstone in the Buzio well has temperatures where chemical compaction could start to occur). However, no clear indication of chemical compaction is observed in the porosity plot, implying that mechanical processes dominate and strengthening the argument that the overpressures inferred using Eaton's method (Fig. 4A and B) are real.

The analysis of the Paleogene mudstone (Fig. 5C) shows initial porosity around 40% at 8 MPa, dropping significantly to around 6% at 36 MPa. The shallower interval in the Buzio well reveals a predominance of mechanical compaction, while the very low porosity observed in the Dugongo well, below the normal compaction trends, indicates a significant effect of chemical compaction consistent with the temperatures greater than 70 °C.

Fig. 5 also compares exponential curves fitted to the data with the basin compaction trend originally proposed by Sclater and Christie (1980) and experimental data from Mondol (2009) for a specific silt-kaolinite (50-50) mudstone case. Sclater and Christie (1980) estimated a compaction trend for shales for the North Sea Basin; Mondol (2009) argued that the silt-kaolinite experimental dataset provides a good model for mechanical compaction since it is a controlled granular medium with a significant clay mineral component.

We observed that the experimental data trend from Mondol (2009) does fit very well to Cretaceous mudstone trends in the Cachalote and Buzio wells (Fig. 5A and B), further supporting the interpretation that mechanical compaction is the dominant process in these wells. However, the Paleogene mudstone in the Dugongo well (Fig. 5C) shows a much lower porosity at corresponding effective stress than the trend measured by Mondol (2009), implying that chemical compaction is likely to be significant in this interval. In addition, the Paleogene mudstone in the Buzio well has porosities significantly higher than the Mondol (2009) trend, further supporting the interpretation that this mudstone is overpressured.

## 4 Discussion

By using multiple data sources and Eaton's method, which is based on effective stress theory, we identify and predict the overpressured mudstone intervals in three wells in the Rovuma Basin. Well test measurements (Fig. 4) in sandstone intervals were used to contrast with predicted pore pressure in mudstones, using Eaton's method.

In the Cachalote and Buzio wells, the mudstone intervals suggest that mechanical compaction is the predominant regime and the pore pressure prediction method work well.

For the deeper Dugongo-1 well, the log-based analysis revealed that the Paleogene mudstones are strongly affected by both mechanical and chemical compactions, with chemical compaction being especially evident in the deeper Paleogene (Paleocene) interval. We therefore observe that, although there is formation temperature similarity between the deeper Cretaceous mudstone in the Buzio well and the Paleocene mudstone in the Dugongo well, other factors must have controlled the differences. Depth effects and most probably the clay mineral composition may have played an important role in determining the chemical compaction regime difference between the wells.

The normal pore pressure from well test measurements in the shallower sandstones was similar to pore pressures predicted from Eaton's method (Fig. 4A and B) in adjacent mudstones. This suggests that the method may be applicable for pore pressure prediction in those mudstone intervals.

In Fig. 4 (C), the well test data from Cretaceous sandstones show elevated pore pressures, somewhat higher than the estimated overpressure interpreted using Eaton's method in the overlying mudstones. Even though the well test measurements were reported to be normally pressured, the trend supports the expectation for overpressures in the mudstones of this deeper well.

We also observe that the Mondol (50-50) compaction trendline (based on experimental data) shows a similar trend to the log-based functions estimated for the Cretaceous mudstone in Cachalote and Buzio wells, while the Sclater and Christie (1980) function does not compare closely to the Cretaceous mudstone intervals in the same wells.

## 5 Conclusions

We have shown how pore pressure evaluation in mudstone intervals under mechanical compaction can be effectively performed using Eaton's method, and demonstrated that the predictions give plausible and informative estimates using well log data. Eaton's method was found to be more suitable for analysis of pore pressure for Buzio and Cachalote wells, where mechanical compaction dominates.

For the Paleogene mudstone in the Buzio well, we find a clear absence of overpressure. Cretaceous mudstones both in the Cachalote and Buzio wells are overpressured, and that is confirmed by contrasting the estimated results with the well test pressure measurements in nearby permeable formations which are normally pressured.

However, Eaton's method underestimates the pore pressure in the Dugongo mudstone, and that is due to a predominant effect of mudstone diagenesis.

In evaluating the compaction trendlines, we see low porosity ranges in the Dugongo well, therefore confirming the chemical compaction effect in that mudstone interval, while in Buzio and Cachalote the temperature effect is negligible.

This study is valuable because it gives an insight into mudstone compaction analysis and pore pressure prediction for the Rovuma Basin, which may be useful in other frontier basin settings.

Comparison of compaction trends in these exploration wells to the experimental data of Mondol (2009) for an artificial 50-50 blend of silt and kaolinite also proved to be valuable, since this experimental dataset is only affected by mechanical compaction.

In future, it would be useful to develop a regional analysis using multiple well data to develop a better understanding of the regional pressure and compaction regimes for different tectonic settings in this under-developed basin. We also hope to extend the analyses to look at sandstone lithologies and to assess the effects of chemical compaction for higher temperature intervals, for example applying the method suggested by Ramdhan and Goulty (2017).

## Declarations

### Author contribution statement

O. Nhabanga: Conceived and designed the experiments; Performed the experiments; Analyzed and interpreted the data; Contributed reagents, materials, analysis tools or data; Wrote the paper.

P. Ringrose: Conceived and designed the experiments; Performed the experiments; Analyzed and interpreted the data; Wrote the paper.

### Funding statement

This work was supported by the EnPe (the Norwegian Programme for Capacity Development in Higher Education and Research for Development within the Fields of Energy and Petroleum).

### Competing interest statement

The authors declare no conflict of interest.

### Additional information

No additional information is available for this paper.
